# Cardanol-Derived Epoxy Resins as Biobased Gel Polymer Electrolytes for Potassium-Ion Conduction

**DOI:** 10.1021/acsapm.2c00335

**Published:** 2022-04-29

**Authors:** Eleonora Manarin, Francesca Corsini, Sabrina Trano, Lucia Fagiolari, Julia Amici, Carlotta Francia, Silvia Bodoardo, Stefano Turri, Federico Bella, Gianmarco Griffini

**Affiliations:** † Department of Chemistry, Materials and Chemical Engineering “Giulio Natta”, Politecnico di Milano, Piazza Leonardo da Vinci 32, 20133 Milano, Italy; ‡ Department of Applied Science and Technology, Politecnico di Torino, Corso Duca degli Abruzzi 24, 10129 Torino, Italy

**Keywords:** biobased epoxy resins, cardanol-based polymers, biobased polymer membranes, succinic anhydride, gel polymer electrolytes, potassium-ion batteries

## Abstract

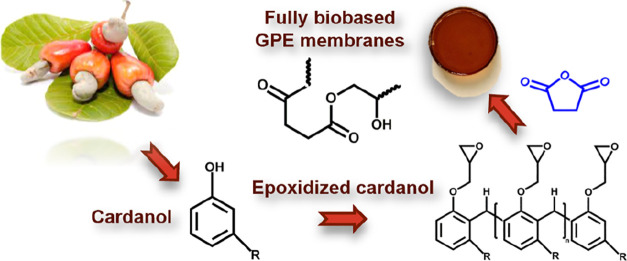

In this study, biobased gel polymer electrolyte (GPE) membranes were developed via the esterification reaction of a cardanol-based epoxy resin with glutaric anhydride, succinic anhydride, and hexahydro-4-methylphthalic anhydride. Nonisothermal differential scanning calorimetry was used to assess the optimal curing time and temperature of the formulations, evidencing a process activation energy of ∼65–70 kJ mol^–1^. A rubbery plateau modulus of 0.65–0.78 MPa and a crosslinking density of 2 × 10^–4^ mol cm^–3^ were found through dynamic mechanical analysis. Based on these characteristics, such biobased membranes were tested for applicability as GPEs for potassium-ion batteries (KIBs), showing an excellent electrochemical stability toward potassium metal in the −0.2–5 V voltage range and suitable ionic conductivity (10^–3^ S cm^–1^) at room temperature. This study demonstrates the practical viability of these biobased materials as efficient GPEs for the fabrication of KIBs, paving the path to increased sustainability in the field of next-generation battery technologies.

## Introduction

In the context of high-performance polymers, epoxy resins represent one of the most widely employed families of thermosetting systems for demanding applications, given their outstanding thermal, chemical, and mechanical resistance, which enables them to be used successfully in a large variety of industrial fields.^1^ However, epoxy resins also exhibit critical issues, mainly associated with the toxicity of some of their precursors, which are typically derived from fossil resources.^2,3^ Within this context, vegetable oils have been considered as a cheap source of potentially useful functional monomers and oligomers to be explored as promising candidates for replacing fossil-based epoxy resins.^4−14^ In particular, cardanol, a highly abundant, nonedible and renewable byproduct extracted from the shell of the cashew nuts, has also been investigated for the production of biobased epoxy resins.^15−17^ On the other hand, in order to obtain fully biobased systems, research efforts in the direction of increased materials sustainability have focused on the development of alternative systems to replace the most commonly used oil-based curing agents, namely amines, amides, hydroxyls, acid anhydrides, phenols, and polyphenols.^18,19^ In this field, particular attention has been given to modified plant oils, biobased acids and anhydrides, biobased amines, biobased phenols, rosin acids, terpenes, and lignin.^20−22^

In view of the main principles and goals of sustainable development, the design and synthesis of such advanced, fully biobased polymers is a prerequisite for their application in technological areas where both high performance and enhanced sustainability set the requirements. In this respect, growing interest is nowadays devoted to the world of rechargeable batteries,^23,24^ whose market penetration is experiencing a significant acceleration not only in portable electronics and sustainable mobility but also in the field of large-scale electricity storage in power plants connected with photovoltaic or wind power stations. In these sectors, safety is a crucial and still an unsolved aspect, as the vast majority of commercial (lithium-based) batteries still work with electrolytes obtained using flammable and, in some conditions, even explosive organic solvents mixed with salts.^25,26^ This has forced the scientific community to investigate the use of polymer matrices for the preparation of quasi-solid electrolytes (where the electrolyte solution is trapped in a cross-linked polymeric matrix)^27−30^ and fully solid ones (in which the mobility of lithium ions is exclusively guaranteed by the segmental movement of polymer chains in the amorphous phase).^31−33^ Most of these polymeric systems are based on poly(ethylene oxide) (PEO) of petrochemical derivation due to its favorable electrochemical stability, ability to reversibly complex metal cations (starting from lithium ions), and its processability aimed at obtaining quasi-solid and solid electrolytes.^34−36^

While lithium-based batteries research rapidly proceeds, the limited lithium reserves (0.0017 wt % in Earth’s crust) and its uneven geographical availability overall lead to inevitable increases in raw material costs, which can typically be sustained in the case of high-tech devices (where commercial costs are already high) or if a suitable recycling chain is implemented to recover and reuse lithium.^37,38^ In parallel, postlithium batteries are also being considered worldwide, especially for those applications where the battery size is large, and the cost must be kept very low, for example, in the case of electrochemical energy storage units to be integrated into buildings or into renewable energy-based power stations. Our group has recently started working on potassium-ion batteries (KIBs), given the cheap and abundant raw material (2.09 wt % in Earth’s crust), its favorable geographical distribution, and its intriguing electrochemical properties (K^+^/K has a potential of −2.93 V vs standard hydrogen electrode, a value close to that of Li^+^/Li, −3.04 V).^39−41^ Also, potassium ions show a weaker Lewis acidity than that of lithium-based counterparts, accompanied by a much smaller Stokes’ radius (i.e., 3.6 vs 4.8 Å) in propylene carbonate, thus guaranteeing a higher ionic conductivity in the liquid electrolyte.^42−44^ Overall, potassium ions can be successfully used as charge carriers between the anode and cathode in KIBs, following the same “rocking-chair” mechanism that has brought lithium-ion battery to the current worldwide success. In view of their potential market penetration, KIBs must target the same safety conditions as the lithium-based counterpart; thus, some very preliminary literature articles are appearing with polymer electrolytes replacing liquid ones.^45−47^ Also, preliminary studies are often carried out in half-cells using potassium metal as the anode (that could as well represent a good choice at the market level considering its low cost). However, its high reactivity further requires an electrolyte system able to keep the device stable with time. One of the major challenges for KIB’s applicability is, in fact, the occurrence of severe side reactions between the electrolyte and electrodes, which result in an unstable solid–electrolyte interphase, and thus a low coulombic efficiency. Additionally, the poor solubility of potassium salt in the traditional ester electrolyte has led to the use of ether solvents.^48^ Hence, designing suitable electrolytes is necessary for the development of efficient and competitive KIBs.^49,50^ To address these issues, in this article, we propose gel polymer electrolyte (GPE) matrices obtained from biobased epoxy resins derived from cardanol and crosslinked with cyclic anhydrides as curing agents, able to be activated by an electrolyte based on potassium salts and showing promising electrochemical performance in view of KIB’s applications. In particular, an electrochemical stability toward potassium metal and a suitable ionic conductivity at room temperature could be achieved as key milestones for future investigations aimed at integrating the proposed bioderived GPE in lab-scale potassium-based secondary battery prototypes.

## Experimental Section

### Materials

Cardanol-derived epoxy novolac resin NC547 was purchased from Cardolite (see Figure S1 in the Supporting Information for the chemical structure), whereas hexahydro-4-methylphthalic anhydride (PAn), glutaric anhydride (GAn), and succinic anhydride (SAn) were bought from Sigma-Aldrich. Triazabicyclodecene (TBD), potassium (cubes in mineral oil, 99.5% trace metal basis), and KPF_6_ (99.5% trace metal basis) were purchased from Merck. Battery grade ethylene carbonate (EC) and diethyl carbonate (DEC) were bought from Solvionic.

### Characterization

Nonisothermal differential scanning calorimetry (DSC) analyses were performed with a DSC 823e Mettler-Toledo instrument under nitrogen atmosphere. The uncured samples (∼5–10 mg) were subjected to a thermal cycle from 25 to 300 °C with different heating rates (β = 5, 10, 15, and 20 °C min^−1^) to determine the curing temperature of the biobased epoxy system, the latter being related to the exothermic peak temperature of the crosslinking reaction (*T*_p_).

Ozawa and Kissinger–Akahira–Sunose (KAS) methods were used to calculate the apparent kinetic activation energy of the curing process (*E*_a_) according to eqs 1 and 2, respectively.
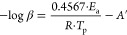
1
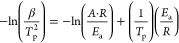
2where *A*′ and *A* are the preindex factors of the Ozawa and KAS methods, respectively, and *R* is the universal gas constant.

Additionally, DSC analyses on samples treated at the selected curing temperature for different curing times (0–120 min) were performed with a thermal cycle from 25 to 300 °C at 10 °C min^–1^ to evaluate the curing kinetics.

Fourier-transformed infrared (FTIR) spectra were recorded using a Thermo Nicolet Nexus 670 instrument. Measurements were performed in transmission mode on solid films deposited on KBr discs, recording 64 accumulated scans at a resolution of 4 cm^–1^.

Thermogravimetric analysis (TGA) was performed with a TA Instruments Q500 under air atmosphere. The cured samples (∼15–20 mg) were subjected to a thermal cycle from 25 to 800 °C at a heating rate of 20 °C min^−1^ to assess the thermo-oxidative stability of the biobased epoxy systems.

To evaluate the extent of crosslinking after curing, gel content measurements were performed by soaking the samples in tetrahydrofuran (i.e., a good solvent for both biobased epoxy resin and anhydrides), subsequently allowing them to dry in vacuum and finally measuring the mass of the dried undissolved material. This procedure was repeated at increasing soaking times. After 24 h, the mass of the dried undissolved materials was found to be constant. Accordingly, the gel fraction (GEL %) was calculated based on eq 3
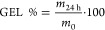
3where *m*_24h_ and *m*_0_ represent the weight of the recovered gel after 24 h soaking and of the pristine samples, respectively.

Dynamic mechanical analysis (DMA) in shear mode was performed with a Mettler-Toledo DMA/SDTA 861e on sample disks with a diameter of 6 mm and a thickness of 2 mm to determine the linear viscoelastic region (LVR) and the thermal transitions of the obtained materials. In the former case, DMA measurements were performed in a strain sweep configuration (1 Hz, strain between 0.1 and 200 μm) at 25 °C. In the latter case, temperature sweep tests (from −50 to 140 °C with a heating rate of 3 °C min^–1^) were conducted at 1 Hz and a deformation amplitude within the LVR (1 μm). From DMA analysis in the temperature sweep configuration, the crosslinking density ν (moles of crosslinking sites per unit volume, mol cm^–3^) was calculated through eq 4
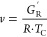
4where *T*_C_ is the characteristic temperature, and *G*_R_^′^ is the shear storage modulus in the rubbery plateau at *T*_C_. *T*_C_ was set at 100 °C (373 K) for all the samples, well above their respective glass transition temperature.

The electrolyte uptake ratio (EUR) was measured for each biobased epoxy membrane at room temperature and under dry atmosphere by swelling each membrane with a standard KIB liquid electrolyte, namely KPF_6_ 0.8 M in EC/DEC 1:1. Each sample was weighted at different time intervals, and EUR values were determined for each measurement through eq 5
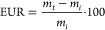
5where *m*_*i*_ and *m*_*t*_ represent the weight of the dry membrane and of the membrane swelled for time *t*, respectively.

The rheological properties of the cardanol-based membranes both in the dry and swollen (with a EC/DEC 1:1 v/v solution) state were measured using a Discovery DHR2 stress-controlled rheometer (TA Instruments) equipped with a 20 mm diameter stainless steel plate and a Peltier plate temperature controller (parallel-plate geometry). In particular, the LVR was measured by varying the shear stress amplitude from 1 to 10^4^ Pa at a constant frequency of 10 Hz. All experiments were carried out at a constant temperature of 25 °C and applying an active normal force on the specimen (10 N for both dry and swollen samples) for the entire duration of the measurements. Linear sweep voltammetry (LSV) was carried out at room temperature using two ECC-Std electrochemical cells by EL-Cell GmbH, able to apply a mechanical load (up to 50 N) on the 18 mm-diameter plunger, ensuring an adjustable, reproducible, and homogeneous pressure on the electrodes. One cell was used to detect the cathodic branch of the LSV measurement; thus, the swollen membrane was sandwiched between potassium and copper foils to be tested from its open-circuit voltage (OCV) down to −0.2 V. Instead, the other cell was assembled according to the following architecture: potassium|swollen membrane|stainless steel. The potential scan for the latter was set from OCV to 5 V to record the anodic branch of the LSV curve. Both measurements were conducted at a scanning rate of 0.1 mV s^–1^. The electrochemical workstation used was the VSP-3e model by BioLogic Sciences Instruments.

The ionic conductivity (σ) values of the swollen GPE membranes were measured by electrochemical impedance spectroscopy (EIS), assembling ECC-Std electrochemical cells, where the swollen membrane was sandwiched between two stainless steel plates. The cell was kept into a digitally controlled climatic chamber (model MK53 × 10^2^.1 by BINDER GmbH), where the temperature was decreased from 50 to 10 °C by 10 °C steps. At each step, the temperature was kept constant for 1 h and then the EIS measurement was performed, allowing to record the value of electrolyte resistance (*R*_b_), at the high-frequency intercept, at that temperature. σ values were calculated through eq 6
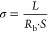
6where *L* is the sample thickness, and *S* is its area.

### Preparation of Biobased Epoxy Membranes

Biobased membranes were prepared using a cardanol-derived epoxy resin (from now on referred to as NC547) and three different cyclic anhydride curing agents: GAn, SAn, and PAn, the latter used as a reference system given its wide application in conventional oil-based epoxy systems.^51−54^ A given amount of NC547 was weighed in a beaker and preheated to 100 °C using a hot plate in order to reduce its viscosity and make the mixing process easier. Then, the selected anhydride was added to the NC547 resin and magnetically stirred until a homogeneous mixture was obtained. The composition was fixed at an anhydride/epoxy group molar ratio equal to 1. Finally, the catalyst (TBD) was added to the mixture in an amount equal to 5 wt % with respect to the moles of epoxy functionalities, and magnetic stirring was continued until a complete dissolution in the blend was achieved. The obtained viscous liquid was deposited by blade coating (K control coater by RK Printcoat Instruments) on a Teflon sheet and transferred into an oven for the curing step (45–90 min, depending on the system) at 160 °C (this temperature was selected based on DSC analyses of the curing process, as detailed in the following).

## Results and Discussion

### Crosslinking of Cardanol-Derived Epoxy Resins with Cyclic Anhydrides

**Scheme 1:**
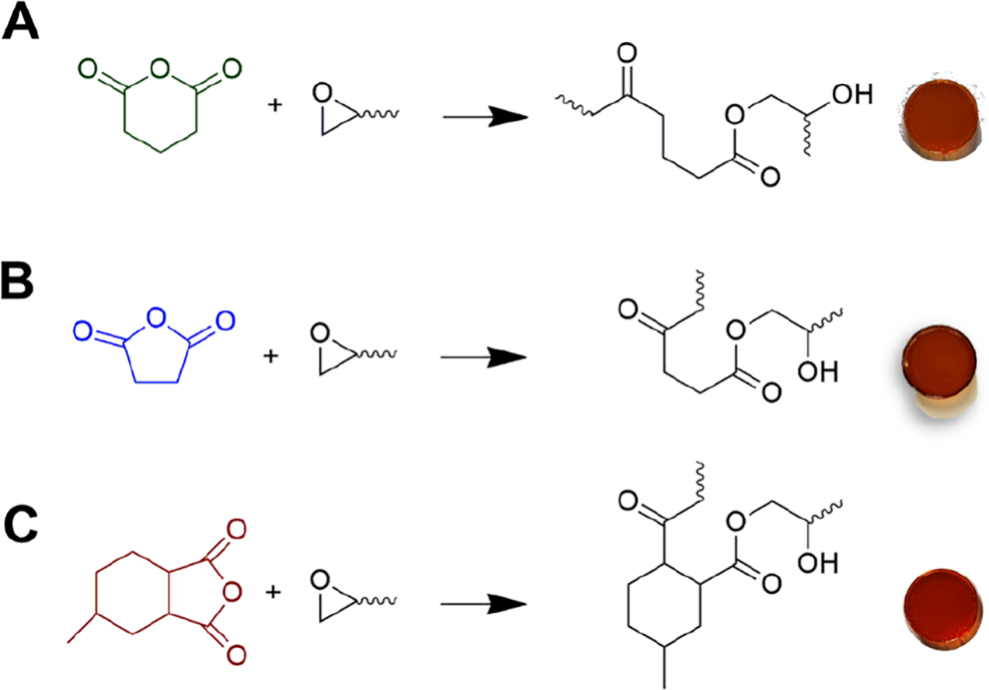
Reaction Mechanism of the Epoxy Ring Opening in NC547 with the Different Anhydrides: (A) GAn, (B) SAn, and (C) PAn Photographic Images of Representative Membranes are Also Reported

To obtain the solid biobased GPE membranes, the cardanol-derived epoxy resin NC547 was crosslinked with three different cyclic anhydrides (GAn, SAn, and PAn) via a ring–opening reaction (Scheme 1).

**Figure 1:**
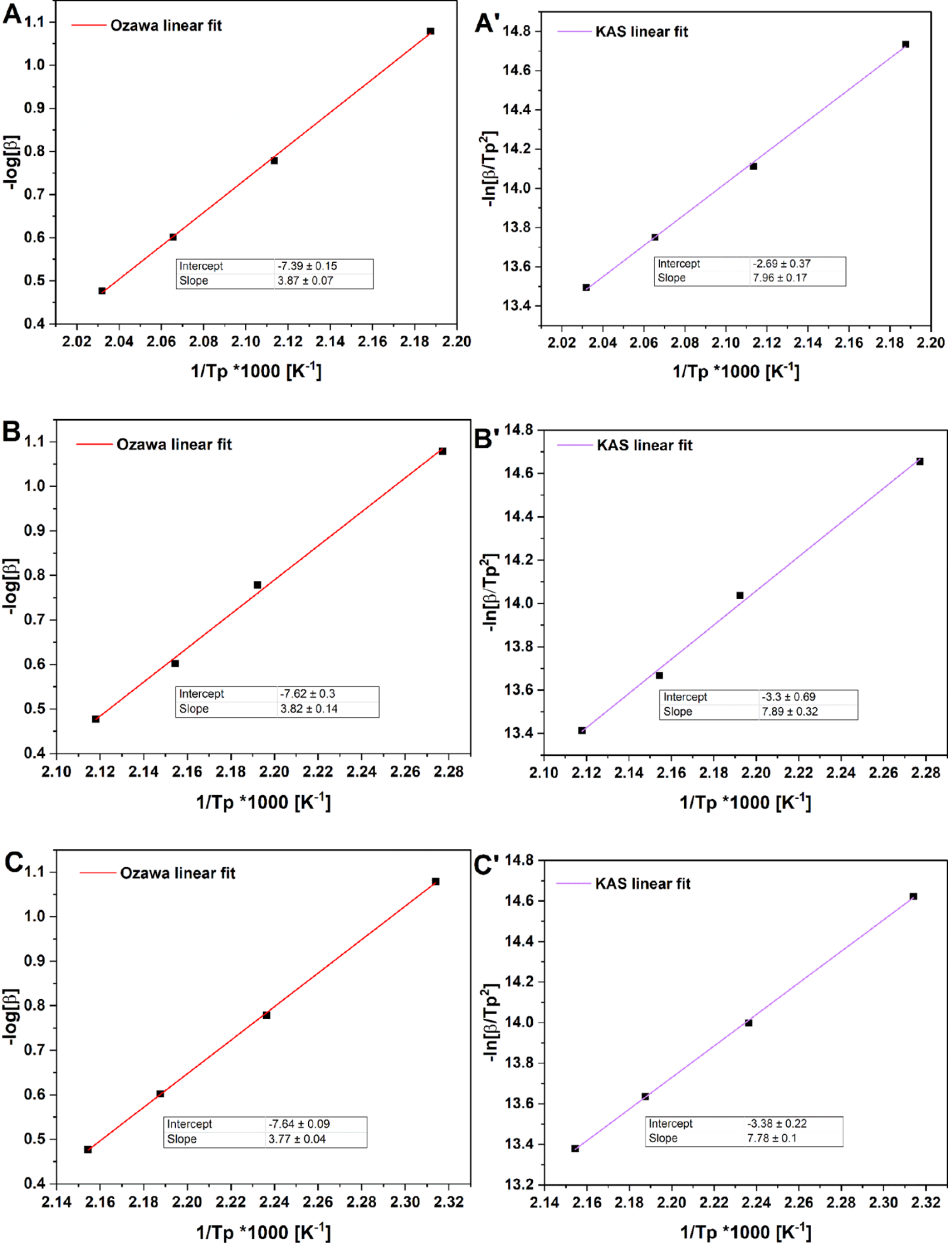
Linear regression curves based on the Ozawa and the KAS methods of (A,A′) GAn-, (B,B′) SAn-, and (C,C′) PAn-based epoxy systems.

A preliminary study on the curing process was conducted by means of DSC analyses to assess the optimal curing temperature and time for each biobased epoxy/anhydride blend. Measurements were performed at different heating rates (β = 5, 10, 15, and 20 °C min^–1^) to determine the *T*_p_ of each system, this parameter being related to the exothermic peak of the crosslinking reaction. From DSC analyses, the *E*_a_ of the curing process (Table 1) was calculated according to the Ozawa (eq 1) and the KAS (eq 2) methods; in Figure 1, the corresponding linear regression curves are reported, while the DSC thermograms are reported in Figure S2 in the Supporting Information.

**Table 1:** Values of *E*_a_ of the Crosslinking Reaction for the Different Cardanol-Derived Epoxy/Anhydride Systems According to the Ozawa and the KAS Methods

composition	*E*_a-Ozawa_ [kJ mol^–1^]	*E*_a-KAS_ [kJ mol^–1^]
NC547-GAn	70.4	66.1
NC547-SAn	69.5	65.6
NC547-PAn	68.5	64.6

These two methods are based on the assumption that the maximum rate of the curing reaction occurs at the peak temperature of the exothermic signal recorded by DSC measurements.^55,56^ As shown in Table 1, the *E*_a_ values for all biobased epoxy systems are relatively comparable with each other irrespective of the curing agent used, indicating that the nature of the cyclic anhydride does not influence significantly the kinetics of the crosslinking reaction. In addition, the relatively low values of *E*_a_ suggest that all systems react rapidly to lead to the formation of the cured network.^57,58^

As expected, the *T*_p_ of each system is closely related to the heating rate β used during the DSC scan (Figure S2). Indeed, as β increases, the onset temperature of the curing process and the associated peak temperature *T*_p_ move toward higher values as a result of the reduced time allowed for the system to react. Finally, the higher the heating rate, the larger the d*H*/d*t* ratio of the systems as a consequence of the increase in the thermal inertia and the associated inhibition of the curing reaction.^59^ Based on these considerations, for all biobased epoxy systems an optimal curing temperature of 160 °C was selected.

**Figure 2:**
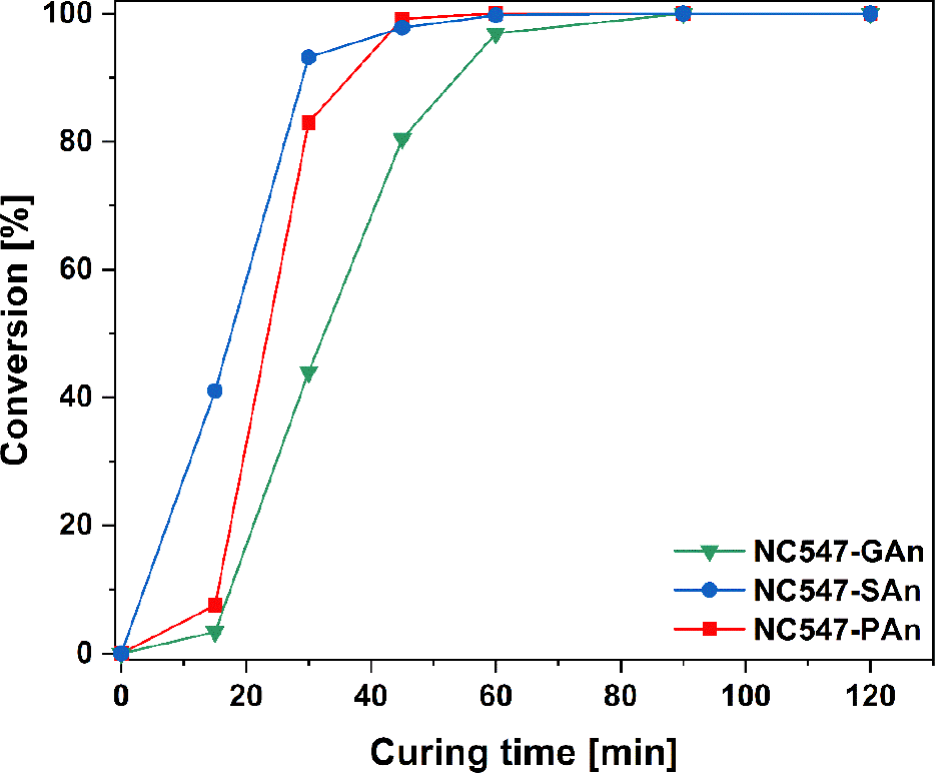
Conversion (%) of the curing reaction of NC547/GAn, NC547/SAn, and NC547/PAn blends at increasing curing time (0–120 min).

Additionally, the kinetics of the curing process was studied via DSC by monitoring the evolution of the exothermic peak in the thermograms associated with the curing reaction during increasing curing times at the curing temperature selected (i.e., 160 °C). The conversion curves of the curing process versus curing time for the three biobased GPE membrane materials are reported in Figure 2.

**Figure 3:**
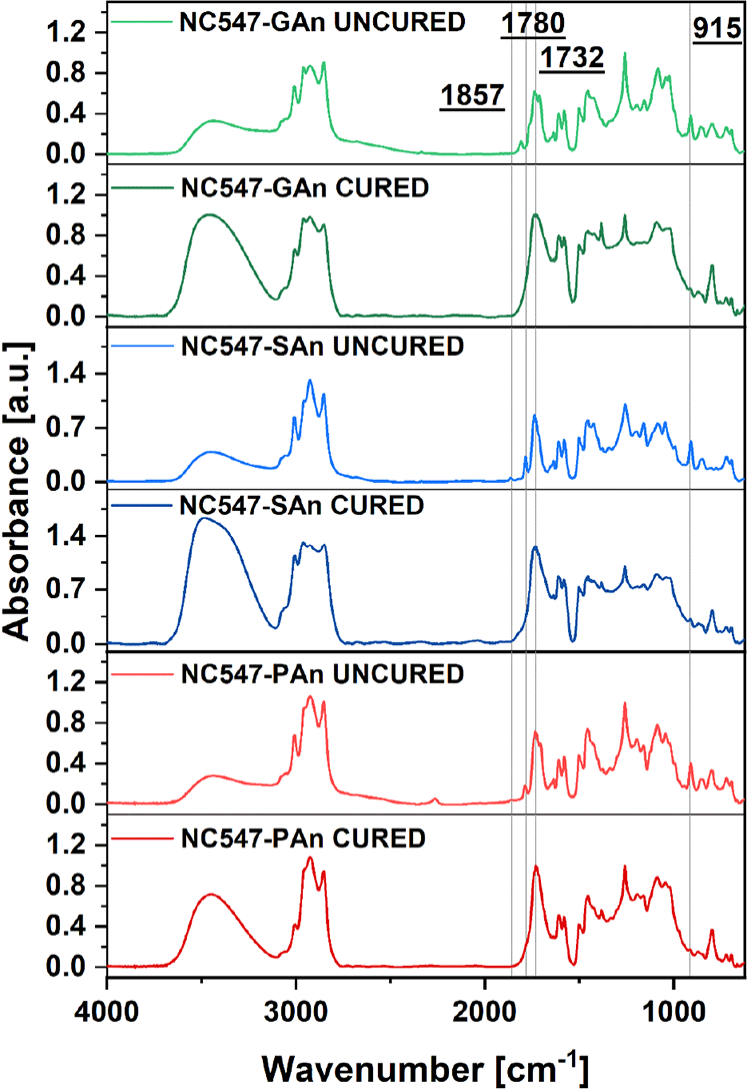
FTIR spectra of uncured and cured GAn-, SAn-, and PAn-based epoxy systems; spectra were normalized with respect to the signal peaked at 1260 cm^–1^, corresponding to the C–O stretching of the benzene ring of NC547.

For all systems, the curing process was found to be fully completed within 90 min of heat treatment at 160 °C, independently of the curing agent. Interestingly, GAn-based membranes were characterized by slower curing kinetics, likely due to the lower reactivity of GAn with respect to SAn and PAn.^60,61^ On the contrary, SAn-based membranes exhibited the fastest conversion rate, yielding complete curing already after ∼45 min of heating. The evolution of the crosslinking reaction was also monitored by means of FTIR spectroscopy, comparing the spectra of the uncured biobased epoxy/anhydride systems with cured ones (Figure 3). In all systems, the disappearance of the peak at 915 cm^–1^ associated with the bending of the oxirane CH_2_–O–CH ring suggests a complete reaction of the epoxy groups in NC547 with the anhydride crosslinker, leading to the formation of ester linkages (as corroborated by the appearance of the carbonyl signal centered at 1732 cm^–1^). In addition, the absence in all cured materials of the signals associated to the stretching of the C=O carbonyl groups of anhydrides centered at 1857 and 1780 cm^–1^ further confirms the full conversion of the epoxy/anhydride reaction, irrespective of the curing agent.

**Figure 4:**
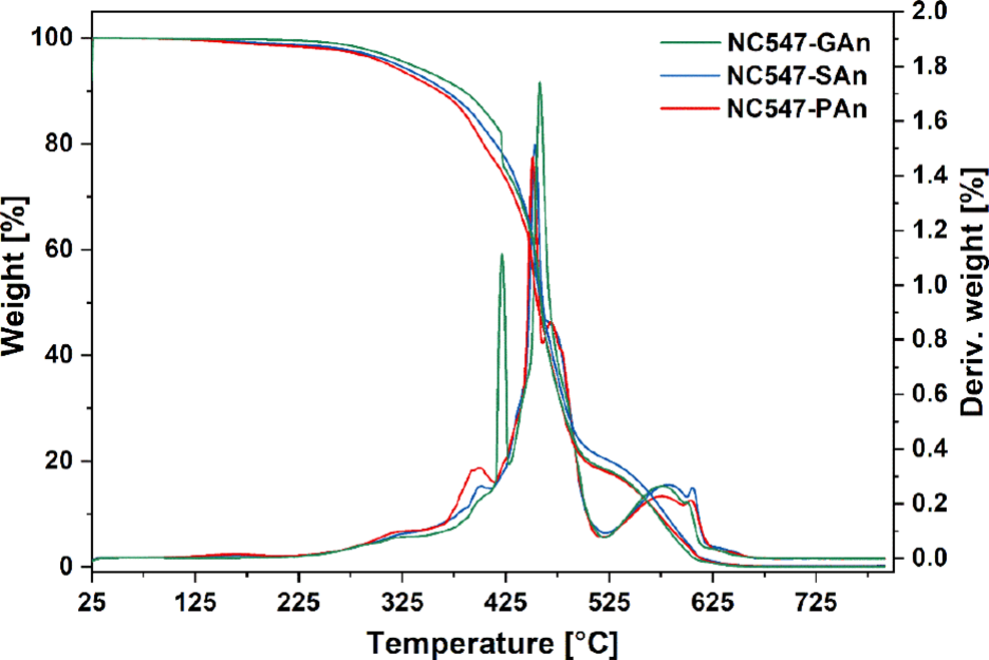
TGA and DTGA curves for the three different NC547-based membranes.

The thermal stability of the cardanol-based epoxy systems was studied by means of TGA analysis in air (Figure 4), considering the degradation temperatures at 5% (*T*_5%_) and 50% (*T*_50%_) of weight loss and at the maximum of the derivative (DTGA) curve (see Table S1 in the Supporting Information). All the cardanol-based membranes show high thermal stability up to 400 °C, independently of the curing agent, with major weight losses only observed in the range between 400 and 600 °C, likely ascribable to the complete disruption of the macromolecular network. Furthermore, at 800 °C, the materials are fully degraded, with char yields of nearly 0%.^62,63^

### Cardanol-Based Epoxy/Anhydride Crosslinked Systems

The extent of crosslinking for the different cardanol-based epoxy/anhydride systems after curing was evaluated by means of gel content experiments, which yielded high values of GEL % (>90%) in all cases (see Table S2 in Supporting Information). This evidence indicates a high degree of conversion of the epoxy/anhydride reaction and a high network connectivity independently of the chemical nature of the anhydride curing agent used.

The mechanical response of all cardanol-based epoxy/anhydride cured membranes was investigated by means of DMA measurements through temperature sweep tests.

**Figure 5:**
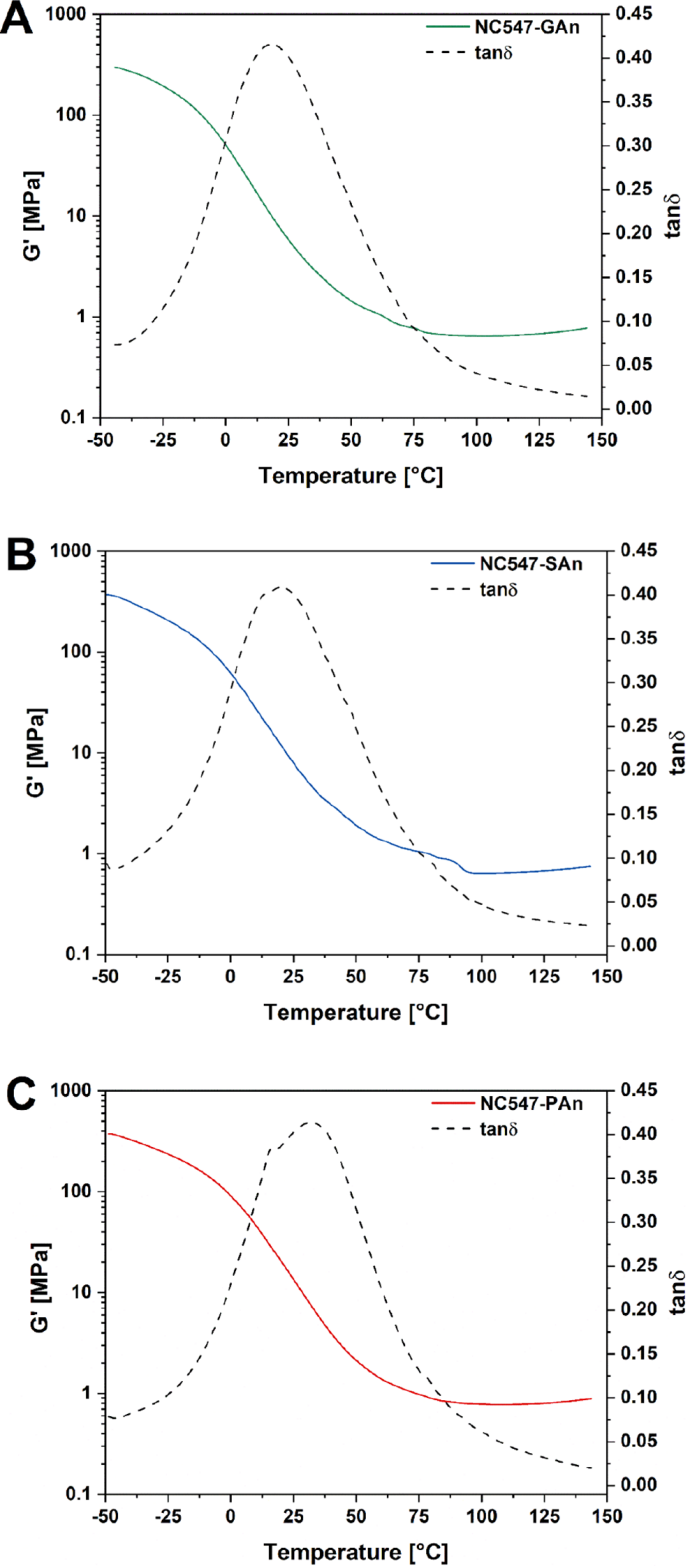
Shear storage modulus (*G*′) and tan(δ) as a function of temperature obtained from DMA in temperature sweep configuration of (A) NC547-GAn, (B) NC547-SAn, and (C) NC547-PAn systems.

As shown in Figure 5 and reported in Table 2, all systems exhibited comparable values of *G*_R_^′^ (∼0.7 MPa) in the rubbery plateau, which resulted to be in line with those reported in the literature for polymer-based electrolytes for battery applications,^29,45,64^ thus suggesting the practical viability of these biobased membrane materials as GPEs (see the Supporting Information for additional mechanical characterization). DMA measurements were also employed to determine their glass transition temperature [*T*_g_, as the temperature of the tan(δ) peak]. Slightly higher values of *T*_g_ were found for PAn-based systems (38 °C) as compared with GAn- and SAn-based ones (20 °C in both cases). As similar ν values were estimated for the different epoxy/anhydride membranes (∼2 × 10^–4^ mol cm^–3^, with a slightly lower value observed in SAn-based systems), this trend could be associated with the characteristic chemical structures of the anhydride crosslinkers employed, with PAn leading to the incorporation of a six-membered ring in the polymer backbone upon reaction with the biobased epoxy resin, thus yielding an increased rigidity of the three-dimensional crosslinked network and, in turn, a slightly higher *T*_g_ value (Table 2).

**Table 2:** Values of *G*_R_^′^ and ν Estimated from the Molecular Theory of Rubber Elasticity and *T*_g_ of the Cardanol-Based Crosslinked Epoxy Systems

composition	*G*_R_^′^ [MPa]	ν [mol cm^–3^]	*T*_g_ [°C]
NC547-GAn	0.642	2.09 × 10^–4^	20
NC547-SAn	0.647	2.07 × 10^–4^	20
NC547-PAn	0.777	2.51 × 10^–4^	38

**Figure 6:**
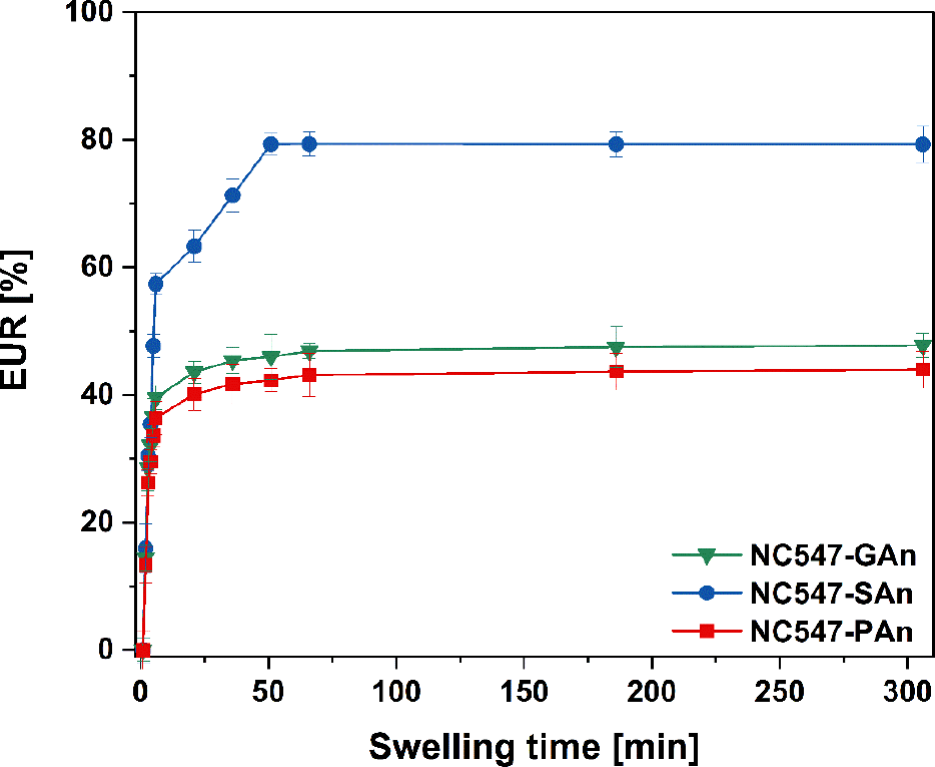
EUR over time with error bands for the three different NC547-based membranes immersed in a standard KIB electrolyte, KPF_6_ 0.80 M in EC/DEC 1:1 (error bars represent standard deviation out of three measurements).

When designing GPEs for battery applications, one of the preliminary requirements to be met is the ability of the polymer network to entrap a liquid electrolyte solution in suitable amounts (≥30% with respect to the polymer weight) without compromising its mechanical stability.^65,66^ The three different cardanol-derived epoxy/anhydride cured membranes demonstrated a remarkable swelling ability when immersed in the electrolyte solution commonly used for KIBs, consisting of KPF_6_ 0.80 M in EC/DEC 1:1. As shown in Figure 6, where the swelling profiles of all membrane materials are reported, a swelling plateau is reached in all systems after ∼45 min of immersion in the electrolyte solution. In particular, in this time interval, PAn-based and GAn-based systems reach a plateau at a maximum EUR value of ∼45%, with no notable differences in the uptake process. Instead, significantly higher mass increase values (80%) are observed in SAn-based membranes.

This trend could be ascribed to the slightly lower crosslinking density of this latter system, which entails intrinsically higher macromolecular chain mobility, ultimately associated with a less compact network and a higher free volume.^67^ On the contrary, the presence of a rigid six-membered ring in the three-dimensional structure of PAn-based materials associated with their slightly higher crosslinking density limits their electrolyte uptake ability, ultimately resulting in lower membrane swelling.

**Figure 7:**
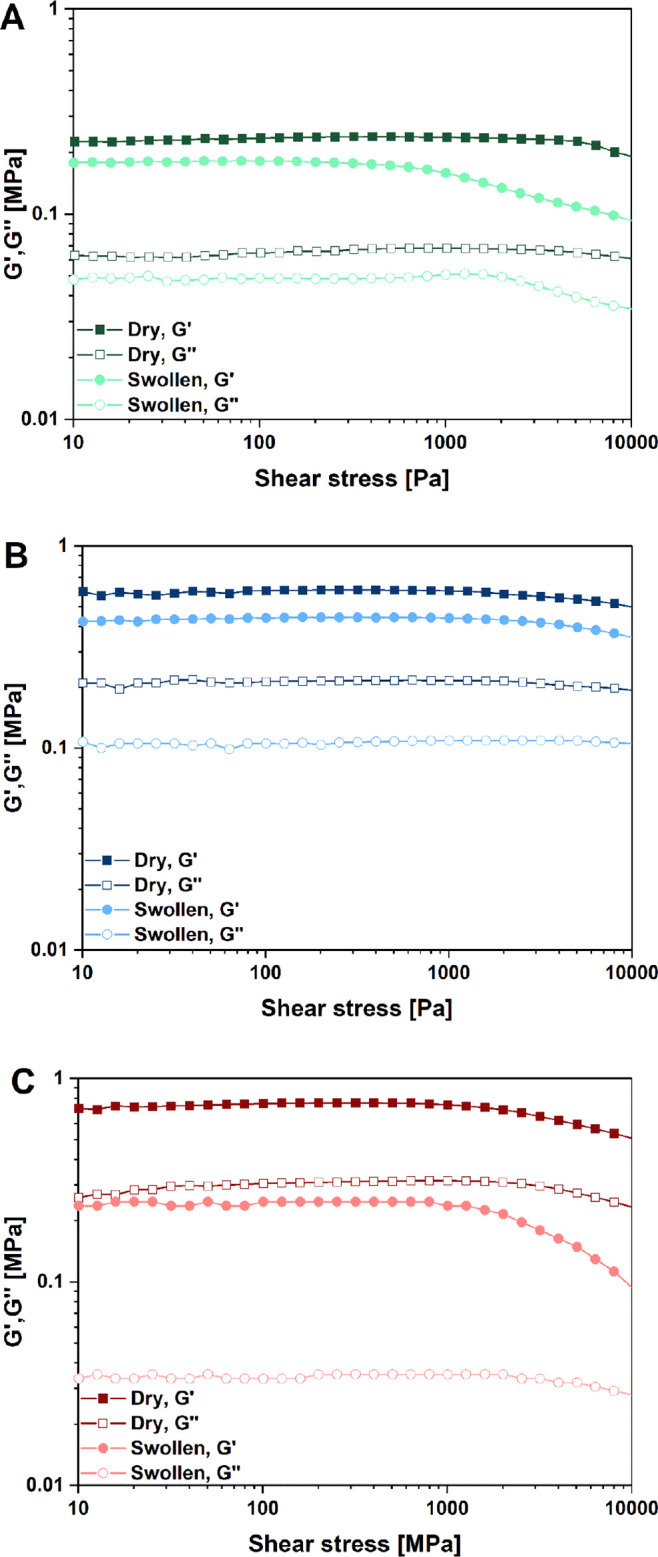
Rheological shear stress sweep tests on (A) GAn-, (B) SAn-, and (C) PAn-based membranes. For all compositions, full symbols indicate *G*′, and hollow symbols indicate *G*″.

The structural stability and the viscoelastic response of the cardanol-based GPEs were investigated through dynamic rheological tests at 25 °C. In particular, the dependence of the shear storage modulus *G*′ and the shear loss modulus *G*″ on the shear stress (τ) was investigated by means of LVR tests. Measurements were carried out on both dried and swollen samples (swelling was performed using the EC/DEC 1:1 v/v solution, based on the outcomes of EUR tests—Figure 6). The results for all the three membrane systems are reported in Figure 7.

Comparing LVR test results of the dry membranes with their unswollen counterpart, it was found that for all three compositions, swelling due to electrolyte uptake led to a decrease of both *G*′ and *G*″ as a consequence of the increased end-to-end distance of the macromolecular chains and the expansion of the porous structure due to solvent intercalation, coupled with its plasticizing action to the polymer chains.^68,69^ As the applied shear stress increased (>1000 Pa), some deviations from linearity of both *G*′ and *G*″ were recorded, leading to a slightly reduced LVR and an earlier onset of irreversible plastic deformation (viz., lower threshold stress values). Notwithstanding these observations, the wide LVR found on all biobased membranes, irrespective of the composition, and their high stress resistance (up to 1000 Pa), appear to be favorable in view of their potential applicability as GPEs for KIBs. Indeed, their excellent mechanical stability could be exploited for device fabrication as no sign of yielding is present in any of the membranes, irrespectively of composition and physical state.^70^ In addition, all the systems show a predominantly solid-like behavior, confirming their full and effective crosslinked nature, which is essential for these GPEs to be used for KIB devices fabrication.^71^

### Electrochemical Characterization of Cardanol-Based Epoxy/Anhydride GPEs

**Figure 8:**
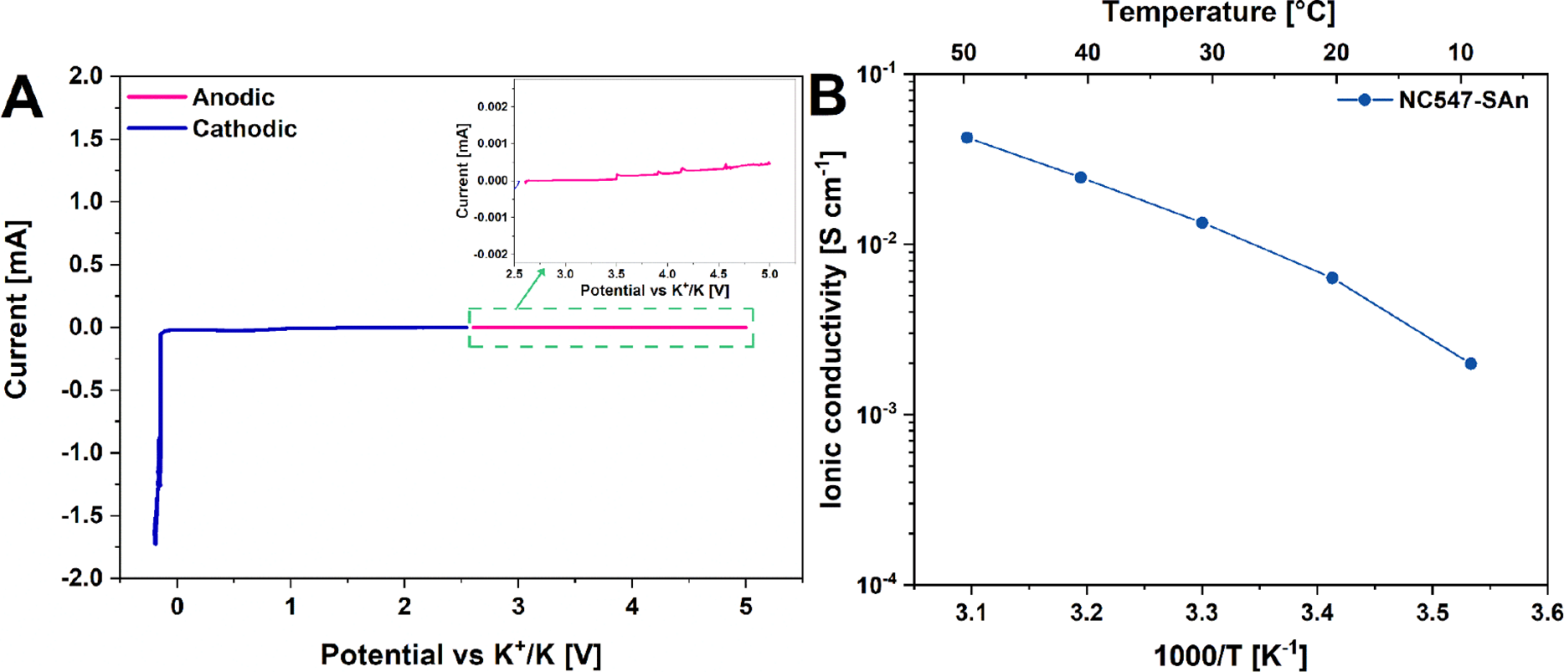
(A) LSV trace for the SAn-based epoxy membrane (activated by swelling in KPF_6_ 0.80 M in EC/DEC 1:1), sandwiched between a potassium and copper foil for the cathodic branch and potassium and stainless steel for the anodic one; cells were tested in the potential range from the OCV to −0.2 V and from the OCV to 5 V, respectively. A zoom of the oxidative stability region is provided. (B) Ionic conductivity data for the SAn-based epoxy membrane (activated by swelling in KPF_6_ 0.80 M in EC/DEC 1:1), measured from 10 to 50 °C and presented as an Arrhenius plot.

Metallic potassium is extremely reactive, and the identification of polymeric matrices able to stabilize this electrode (inhibiting both the growth of metal dendrites and the reactivity of K towards the electrolyte components) is one of the main challenges faced nowadays by the scientific community working in the field of KIBs. In this context, the evaluation of the electrochemical stability window (ESW) represents a fundamental experiment to quantitatively gauge the applicability of a polymeric membrane as GPE and its compatibility with the associated metallic electrode. Accordingly, the ESW of the SAn-based system activated by swelling it in the KPF_6_ electrolyte solution was tested through LSV both in the anodic and the cathodic regime. The resulting current–voltage profile is shown in Figure 8A. Interestingly, the proposed biobased GPE showed a notably wide ESW, with near nil current signal for the whole potential range explored (from −0.2 to 5 V, viz., the one of interest for KIB applications), thus indicating its excellent compatibility with potassium metal. No side reactions were found to occur at any voltage value, being the negative peak revealed at −0.2 V related to the potassium reduction reaction.^65^

Together with the assessment of the electrochemical stability of the GPE in the voltage range of interest for the selected application, another important figure of merit to be considered for gauging its possible use in KIBs is represented by the ionic conductivity of the swollen membrane in a relevant temperature window. In a quasi-solid system such as the one studied in this study, the conduction of ions (K^+^, PF_6_^–^) occurs both in the liquid solution trapped in the membrane and via ion motion along the polymer chains. The latter is more relevant if the polymer matrix is amorphous.^72^ DSC traces of cured SAn-based membranes did not reveal the presence of any melting/crystallization peak and highlighted one single glass transition, thus confirming the completely amorphous nature of the polymer matrix and the excellent thermodynamic compatibility between SAn and the biobased NC547 epoxy resin (see Figure S5 in the Supporting Information). Based on these considerations, ionic conductivity measurements between 10 and 50 °C were performed, considering this as a plausible temperature range for KIB applications in large-scale energy storage plants.^73,74^ As shown in Figure 8B, the activated GPEs possessed an appreciable ionic conductivity with values ranging between 10^–3^ and 10^–2^ S cm^–1^ when passing from lower (10 °C) to higher temperatures (50 °C), respectively. Interestingly, lower ionic conductivities were found for GAn- and PAn-based membranes over the same temperature interval, in line with their less favorable EUR response as compared with SAn-based systems (see Figure S6 in the Supporting Information). Overall, the ionic conductivity of the SAn sample compares well with those typically found in the literature for GPEs used in KIBs and appears to be amongst the highest encountered in the field for biobased quasi-solid polymer electrolytes.^49^ Indeed, the cardanol-based electrolyte ionic conductivity at room temperature (10^–3^ S cm^–1^) exceeds by one order of magnitude the values usually obtained for relevant quasi solid-state electrolytes previously published in the literature. This is valid also for the well-established PEO-based matrices. As a matter of fact, Fei et al. formulated a PEO/KFSI solid-state polymer electrolyte,^75^ while Jeedi et al. designed a PEO/PVdF/KNO_3_ system;^76^ both of these systems led to ionic conductivity values lower than those reported in this study (1.14 × 10^–5^ and 8.79 × 10^–5^ S cm^–1^ at room temperature, respectively). Indeed, room temperature ionic conductivities in the 10^–3^ S cm^–1^ range are typically sufficient to fabricate working devices, thus confirming the potential viability of these cardanol-derived systems as alternative biobased GPEs for KIBs.^77,78^

## Conclusions

In summary, GPE membranes based on a cardanol-derived epoxy resin crosslinked with three different anhydrides (glutaric, succinic, and hexahydro-4-methylphthalic) were proposed in this study for potential application as biobased quasi-solid polymer electrolytes in KIBs. The optimal curing time and temperature were evaluated by nonisothermal DSC analysis at different heating rates, and the activation energy of the curing process was estimated through the Ozawa and the KAS methods and found to be in the order of 65–70 kJ mol^–1^ for all the three compositions. FTIR spectroscopy confirmed the effective occurrence of the curing reaction via epoxy ring opening, leading to a crosslinked system of high network connectivity, as also supported by gravimetric gel content measurements. DMA measurements evidenced a rubbery-plateau modulus in the 0.65–0.78 MPa range for all systems, with a crosslinking density in the order of 2 × 10^–4^ mol cm^–3^. Swelling tests conducted on these biobased membrane materials evidenced that the highest electrolyte uptake was recorded when using SAn as anhydride curing agent, which yielded a plateau value of an 80% mass increase after ∼45 min of immersion in a typical KPF_6_ electrolyte solution. The same systems showed an excellent electrochemical stability toward potassium metal in the range between −0.2 and 5 V and a suitable ionic conductivity (10^–3^ S cm^–1^) at room temperature. This study provides the first demonstration of cardanol-based epoxy/anhydride systems as quasi-solid polymer electrolyte membranes for potassium metal electrochemical cells and demonstrates their potential viability as efficient biobased GPEs for the fabrication of KIBs, paving the path to increased sustainability in the field of next-generation battery technologies.

## Supporting Information Available

Chemical structure of the cardanol-based resin; DSC traces of uncured samples for curing kinetics studies; FTIR spectra of the cardanol-based epoxy resin and anhydrides used as curing agents; TGA, gravimetric gel content, and mechanical analyses; DSC traces of cured SAn-based samples; and ionic conductivity measurements (PDF)
