# Supplementary material for: Cardanol-Derived Epoxy Resins as Biobased Gel Polymer Electrolytes for Potassium-Ion Conduction
Source: ACS Appl Polym Mater. 2022 Apr 29;4(5):3855–65. doi: 10.1021/acsapm.2c00335 (PMC9112699; doi:10.1021/acsapm.2c00335)
Supplement: Supplementary file 1 — ap2c00335_si_001.pdf [file ap2c00335_si_001.pdf]

Supporting Information

Eleonora Manarin[a,†], *Francesca Corsini*[a,†], *Sabrina Trano*[b], *Lucia Fagiolari*[b], *Julia Amici*[b], *Carlotta Francia*[b], *Silvia Bodoardo*[b], *Stefano Turri*[a], *Federico Bella*[b,*], *Gianmarco Griffini*[a,*]

[a]Department of Chemistry, Materials and Chemical Engineering "Giulio Natta", Politecnico di Milano, Piazza Leonardo da Vinci 32, 20133 Milano, Italy

E-mail: gianmarco.griffini@polimi.it

[b]Department of Applied Science and Technology, Politecnico di Torino, Corso Duca degli Abruzzi 24, 10129 Torino, Italy

E-mail: federico.bella@polito.it

Table of Contents

1. CHEMICAL STRUCTURE OF CARDANOL-BASED EPOXY RESIN S3
2. DSC KINETICS S4
3. FTIR SPECTRA S6
4. TGA S7
5. DMA LINEAR VISCOELASTIC REGION CURVES S8
6. GEL FRACTION..S9
7. MECHANICAL CHARACTERIZATION: UNIAXIAL TENSILE TESTS...........................S10
8. DSC OF CURED BIOBASED EPOXY-SAn MEMBRANES...S11
9. IONIC CONDUCTIVITY S12

1. CHEMICAL STRUCTURE OF CARDANOL-BASED EPOXY RESIN

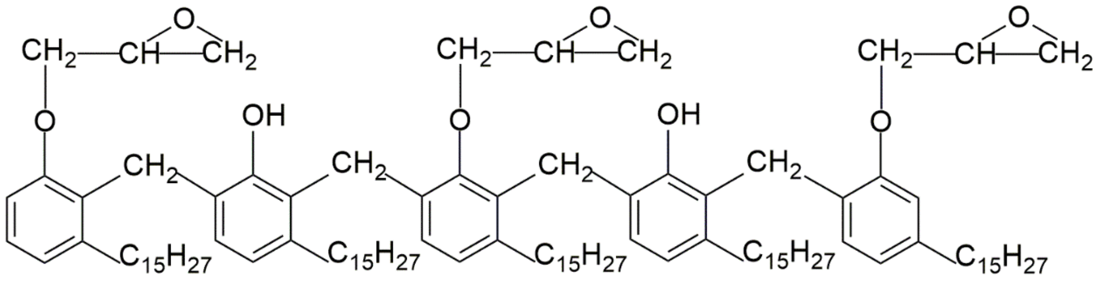

Figure S1. Chemical structure of Cardolite NC547.

2. DSC KINETICS

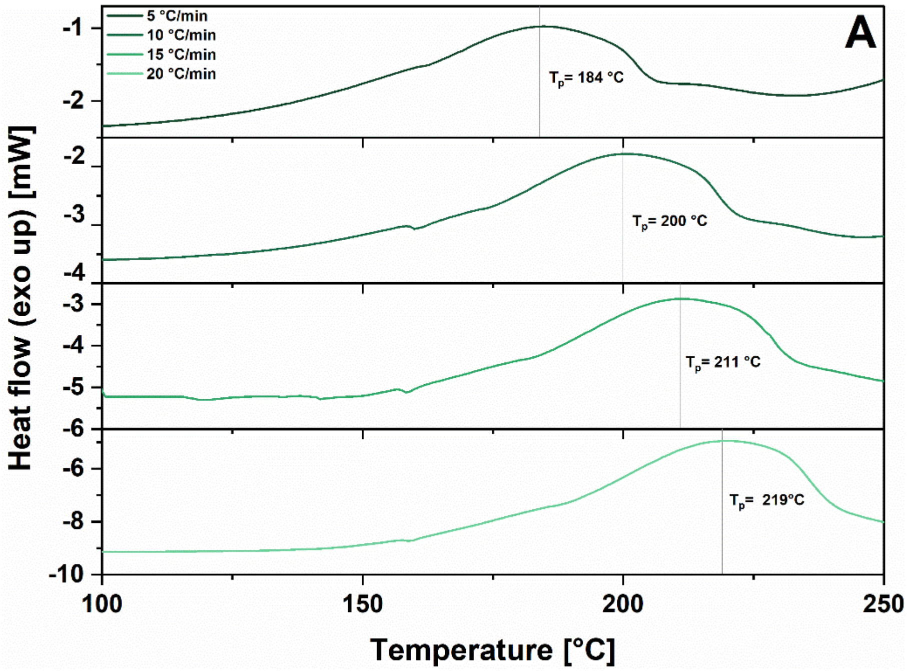

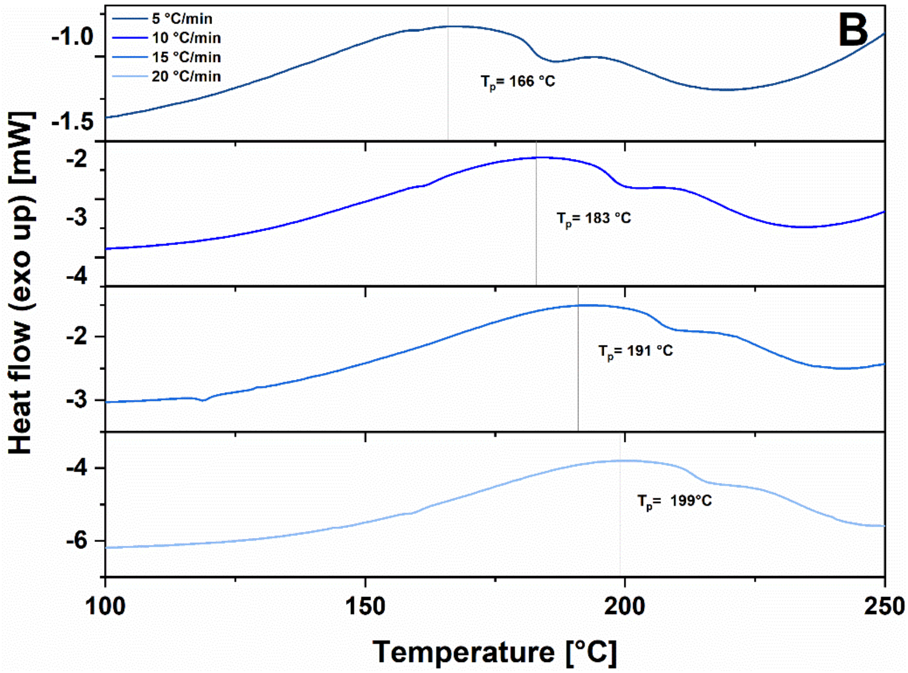

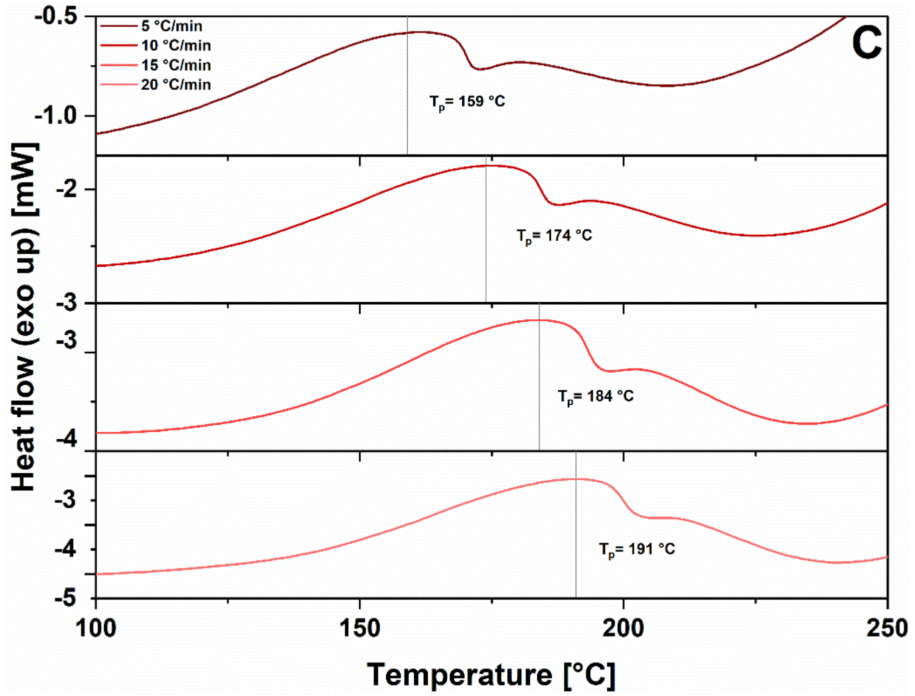

Figure S2. DSC profiles of A) NC547-GAn, B) NC547-SAn and C) NC547-PAn systems, highlighting the curing reaction exothermic peak during heating cycle at various heating rate.

3. FTIR SPECTRA

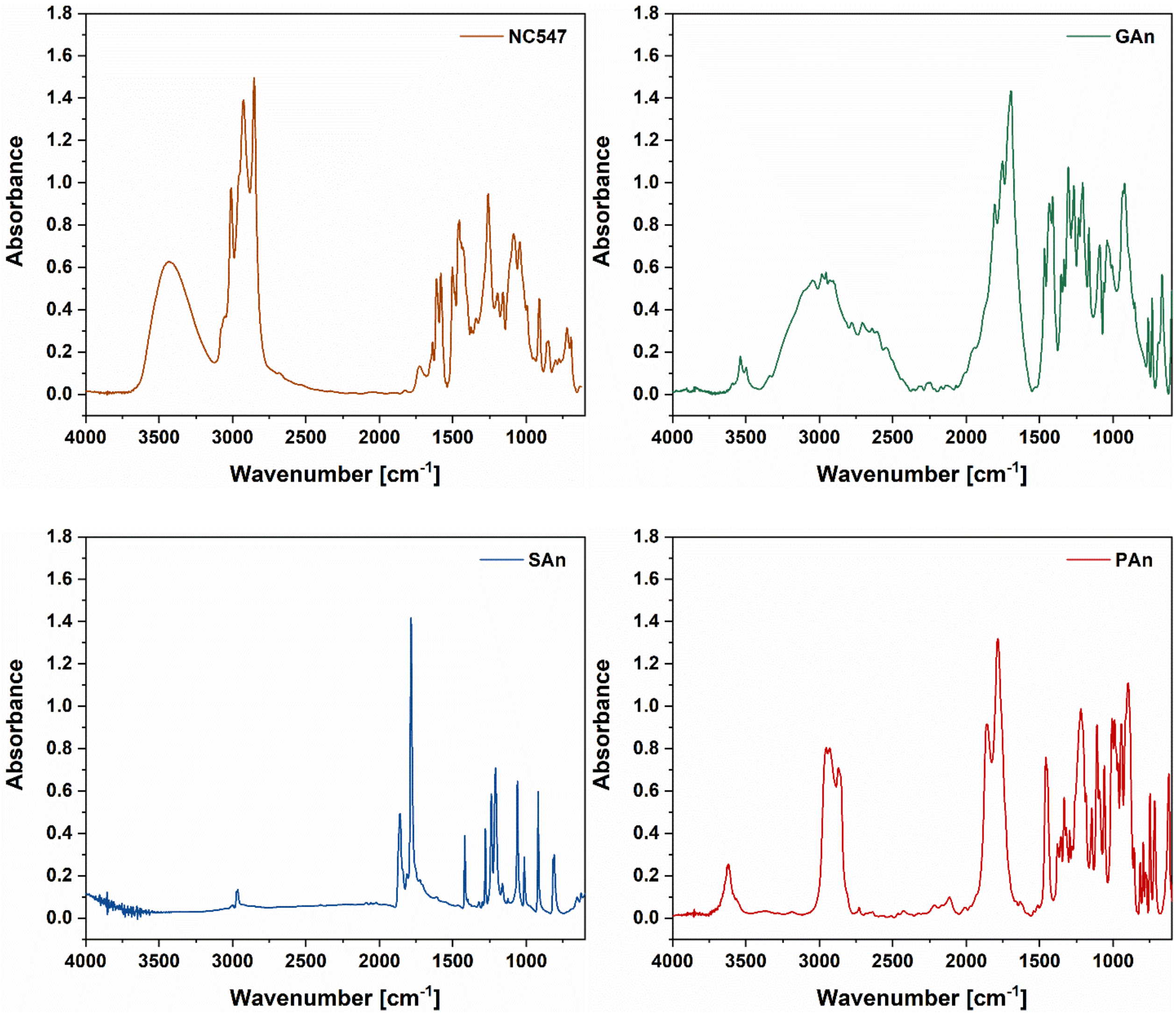

Figure S3. FTIR spectra of Cardolite NC547, GAn, SAn and PAn.

4. TGA

Table S1. Degradation temperatures at 5% ($T_{5\%}$) and 50% ($T_{50\%}$) of weight loss and at the maximum (T_{max}) of the DTGA curves and char yield at 800 °C for the cardanol-derived epoxy crosslinked samples.

**Composition**	**$T_{5\%}$ [°C]**	**$T_{50\%}$ [°C]**	**T_{max} [°C]**	**Char yield [%]**
NC547-GAn	333	459	458	0.04
NC547-SAn	320	459	453	0.16
NC547-PAn	312	455	451	0.10

5. DMA LINEAR VISCOELASTIC REGION CURVES

The shear storage modulus G’ as a function of the strain amplitude for the three cured NC547-based membrane materials is reported in **Figure S4**. As shown, the linear viscoelastic region is evidenced by the shaded area.

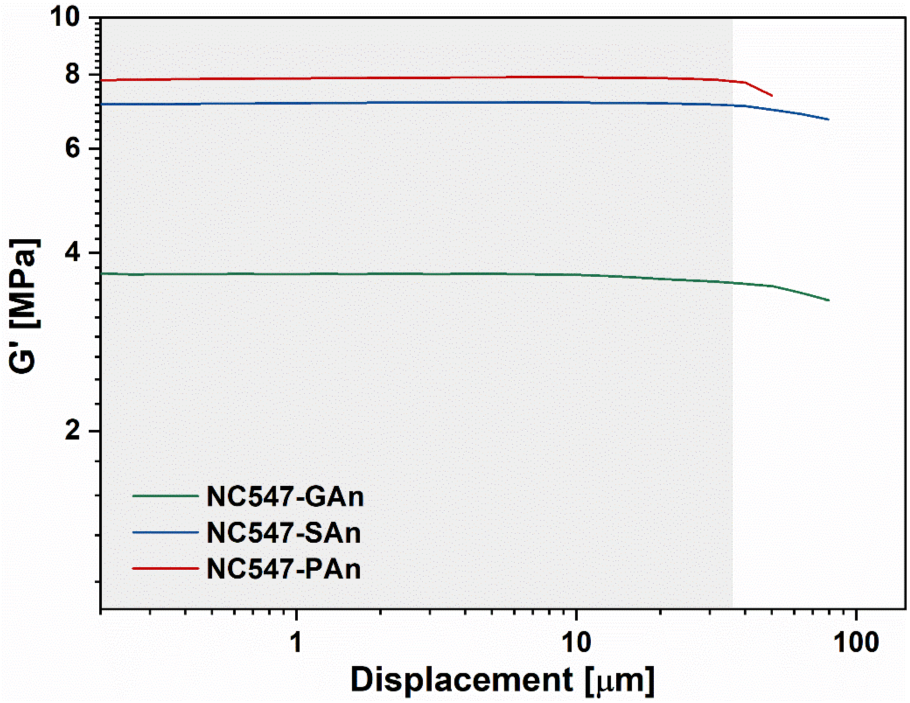

Figure S4. G’ as a function of displacement for the three cardanol-derived epoxy crosslinked samples.

6. GEL FRACTION

Table S2. Values of gel fraction (GEL %) of the cardanol-derived epoxy crosslinked samples.

Composition	GEL %
NC547-GAn	90.4
NC547-SAn	90.2
NC547-PAn	91.0

7. MECHANICAL CHARACTERIZATION: UNIAXIAL TENSILE TESTS

To further corroborate the findings obtained from dynamic mechanical analysis (DMA), uniaxial tensile tests were performed according to ASTM D638-14 on 2 mm thick dumbbell-shaped specimens using a Zwick/Roell BT-FR010TH.A50 dynamometer equipped with a 10 kN load cell, applying a displacement rate of 1 mm/min. Deformation was measured using a long-stroke extensometer.

Three main figures of merits were evaluated: elastic modulus (E_G), deformation at break (ε_r) and ultimate tensile strength (σ_r). Their values for the different materials considered in this work are reported in **Table S3**.

Table S3. Values of E_G, ε_r and σ_r of the cardanol-derived epoxy crosslinked samples.

**Composition**	**E_G [MPa]**	**σ_r [MPa]**	**ε_r [%]**
NC547-GAn	26.47 ± 0.61	0.48 ± 0.13	2.33 ± 0.67
NC547-SAn	33.39 ± 6.39	0.52 ± 0.07	1.98 ± 0.32
NC547-PAn	68.37 ± 19.41	1.52 ± 0.38	3.13 ± 0.46

8. DSC OF CURED BIOBASED EPOXY-SAn MEMBRANES

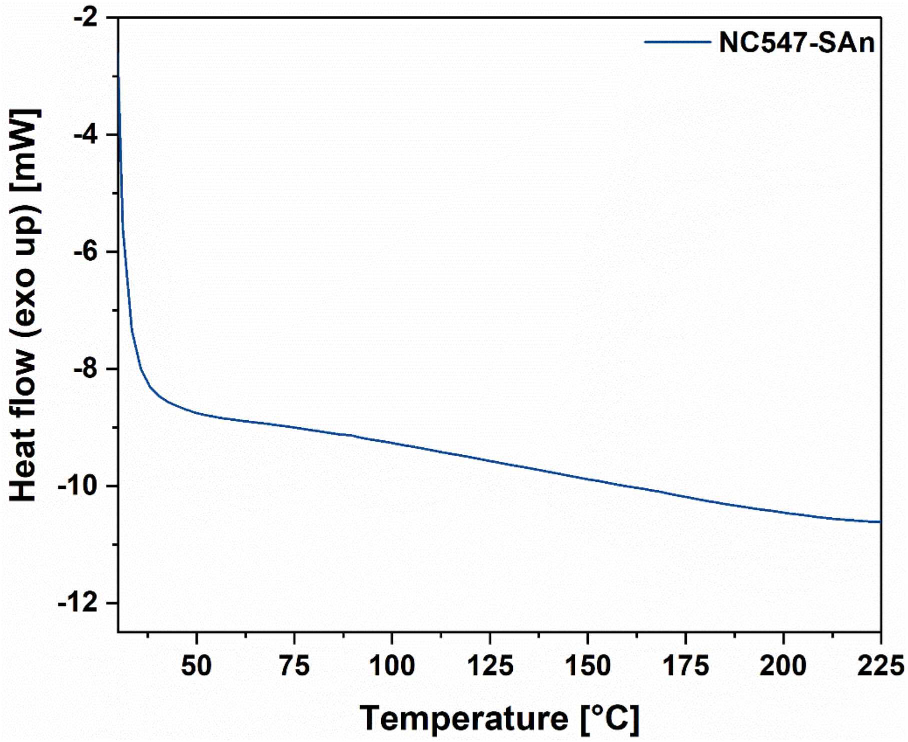

Figure S5. DSC traces of crosslinked epoxy membranes based on SAn as curing agent.

9. IONIC CONDUCTIVITY

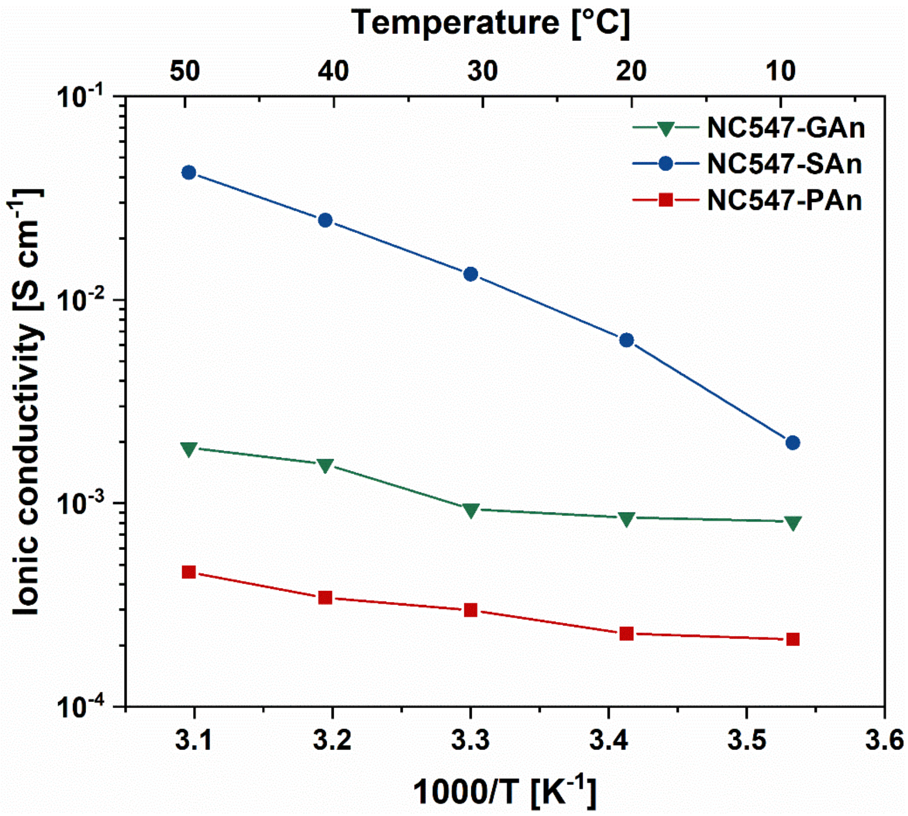

Figure S6. Ionic conductivity of all the three NC547-based membrane swollen in KPF_6 0.80 M in EC:DEC 1:1.
